# Ground Penetrating Radar as a Contextual Sensor for Multi-Sensor Radiological Characterisation

**DOI:** 10.3390/s17040790

**Published:** 2017-04-07

**Authors:** Ikechukwu K. Ukaegbu, Kelum A. A. Gamage

**Affiliations:** Engineering Department, Lancaster University, Lancaster LA1 4YW, UK; k.gamage@lancaster.ac.uk

**Keywords:** ground-penetrating radar, radiological characterisation and multi-sensor data fusion

## Abstract

Radioactive sources exist in environments or contexts that influence how they are detected and localised. For instance, the context of a moving source is different from a stationary source because of the effects of motion. The need to incorporate this contextual information in the radiation detection and localisation process has necessitated the integration of radiological and contextual sensors. The benefits of the successful integration of both types of sensors is well known and widely reported in fields such as medical imaging. However, the integration of both types of sensors has also led to innovative solutions to challenges in characterising radioactive sources in non-medical applications. This paper presents a review of such recent applications. It also identifies that these applications mostly use visual sensors as contextual sensors for characterising radiation sources. However, visual sensors cannot retrieve contextual information about radioactive wastes located in opaque environments encountered at nuclear sites, e.g., underground contamination. Consequently, this paper also examines ground-penetrating radar (GPR) as a contextual sensor for characterising this category of wastes and proposes several ways of integrating data from GPR and radiological sensors. Finally, it demonstrates combined GPR and radiation imaging for three-dimensional localisation of contamination in underground pipes using radiation transport and GPR simulations.

## 1. Introduction

The detection of ionising radiation is critical in fields such as medicine, security and monitoring and decommissioning of nuclear sites and facilities. While every ionising radiation is associated with some level of hazard, highly penetrating neutron and gamma radiations are of particular interest. This is because their high penetrability makes them be both harmful and beneficial; harmful because they pose significant dosage risks to both humans and materials even from far off distances and beneficial because they can be used to detect and image objects located in opaque environments, such as internal body organs [1], buried mines [2], etc. Furthermore, their high penetrability enables them to be detected from stand-off distances, thereby allowing appropriate safety measures to be implemented. Consequently, a wide range of radiological sensors [3,4,5,6] has been developed for stand-off non-destructive detection of both neutron and gamma radiations. These sensors exploit the effects of the interactions (i.e., absorption, scattering and pair production) of these radiations with special materials to detect and image the sources of these radiation, thereby enabling them to be characterised [7].

However, radiation sources exist in an environment referred to as context. This refers to surrounding extrinsic factors that influence the detection, localisation and subsequent retrieval of these sources of radiation. For instance, the context of a stationary source is different from a source in motion because the motion of the source imposes additional challenges to its detection. Similarly, characterising sources located on visible surfaces is different from characterising sources embedded in materials because of the difficulty in obtaining the depth of contamination and the increased influence of background radiation. Furthermore, the relative location of diseased organs, induced with radiation, with respect to other vital organs is critical in proper treatment planning. Other contextual factors include, material and geometry of surrounding objects and weather condition, e.g., rainfall, pressure, etc. [8]. Unfortunately, radiation detectors are unable to measure these extrinsic factors, hence the need for integration with a host of contextual sensors, e.g., visual, ultrasonic, microwave, location, etc. This enables the creation of a holistic view of the environment under investigation, thereby enabling the detection and characterisation of the radioactive source(s) of interest.

Furthermore, the benefits of integrating radiological and contextual sensors in medical imaging have been extensively researched and reported [9]. However, integration of both types of sensors has also resulted in interesting and innovative solutions to challenges in characterising radioactive sources in non-medical applications, such as security, non-proliferation and decommissioning of nuclear sites and facilities. However, this latter category of applications is dominated by the integration of radiation and visual sensors. Furthermore, visual sensors are unable to retrieve contextual information about radioactive wastes located in opaque and hard to access environments commonly encountered in nuclear sites and facilities. These wastes include: contaminated underground pipelines used to transport liquid waste; leaked effluents from such pipes and storage ponds; and radioactive contaminant ingress into porous materials, such as concrete [10,11]. Consequently, there is need to integrate radiation data with contextual data from geophysical sensors such as ground-penetrating radar (GPR).

Therefore, this paper presents a comprehensive review of non-medical applications of the integration of radiological and contextual sensors. Furthermore, it proposes several ways of integrating contextual data from GPR with radiological data in light of the techniques presented in the reviewed works and with particular focus on nuclear decommissioning applications. In addition, it also demonstrates the effectiveness of combined GPR and radiation imaging for three-dimensional (3D) localisation of contamination in underground pipes using radiation transport and GPR simulations. The remaining part of the paper is divided into four sections. Section 2 is a review of the integration of radiological and contextual sensors in non-medical applications with the aim of highlighting the key role played by contextual sensors. Section 3 proposes GPR as a contextual sensor for radiological characterisation of hard to access wastes in nuclear sites. Section 4 presents modelling and simulation of combined radiation and GPR for 3D localisation of contamination in buried pipes. Finally, concluding discussions and future directions are presented in Section 5.

## 2. Integration of Radiological and Contextual Sensors

**Figure 1:**
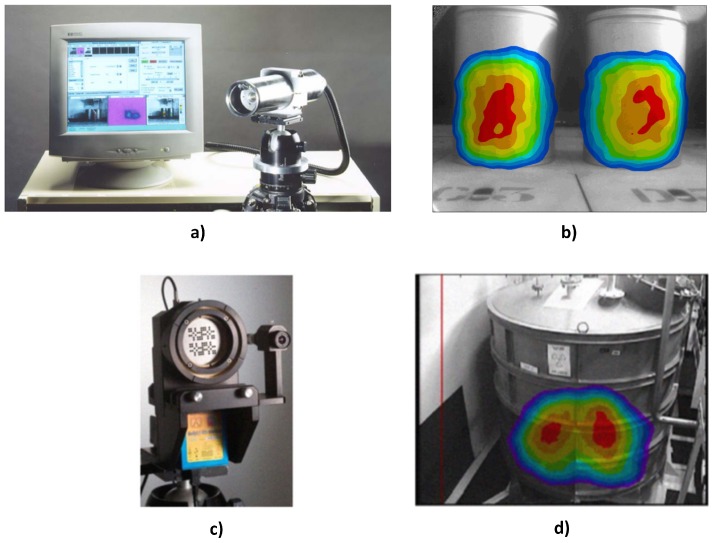
(**a**) CARTOGAM imaging system. The detector head beside the PC is mounted on a tripod [12]. (**b**) Superimposed radiation and visual images from the CARTOGAM [13]. (**c**) GAMPIX gamma camera (small visual camera attached on the left). (**d**) Superimposed radiation and visual images from the GAMPIX camera [4].

The reported techniques used in integrating data from radiological and contextual sensors can be broadly classified into passive and active depending on the integration process and the role played by the contextual data in the final output. In passive techniques, the integration process is not based on any mathematical or logical formalism, and the contextual data only serve as a passive backdrop for the radiation data. A classic example is the superimposing of radiation images over visual images of the scene (Figure 1b,d). On the other hand, active techniques are based on some mathematical or logical model where both the radiation and contextual data actively contribute in determining the final outcome of the integration process. Specific applications of both classes of integration techniques will be described in the following subsections.

### 2.1. Passive Integration of Radiological and Contextual Sensors

Superimposing of the images of the detected sources over a visual image of the environment [3,4,5,12,13] is perhaps the default scheme for combining radiation and contextual data because of its simplicity. This quickly identifies the object(s) to which the radiation sources are attached. Both images could be obtained by the same camera since a gamma detector is sensitive to both gamma and visible photons. For instance, the CARTOGAM gamma camera (Figure 1a) is able to operate in both visible and gamma image mode. During gamma imaging, a thin shutter closes the collimator and prevents the entrance of visible light so that scintillation is only caused by gamma photons penetrating the shutter. To capture visible images, the shutter is opened, and a small lens is remotely placed at the collimator centre. This makes both images be accurately aligned spatially with respect to the imaged scene. However, capturing both gamma and visual images with the same camera increases the overall image acquisition time as both images cannot be captured simultaneously because they require different camera settings [3]. Other cameras, such as [4,5,14], employ a separate visual camera collocated with the detector (Figure 1c). Such configurations are able to take advantage of advances in visual camera technology (e.g., panoramic imaging) and video imaging to give a more realistic and real-time visual rendering of the imaged scene over which the gamma image is subsequently overlaid (Figure 1d). However, the radiation and visual cameras will have to be physically aligned for accurate superimposing of both images.

**Figure 2:**
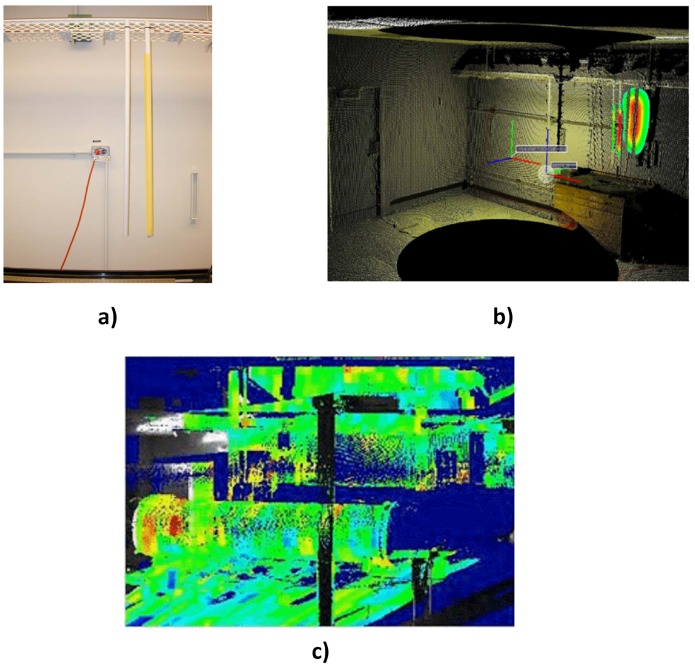
(**a**) Zoomed-in picture of the experimental contaminated environment. The contaminated pipe is indicated in yellow colour [17]. (**b**) Gamma image projected into the 3D LiDAR image of the scene in (a) [17]. (**c**) Gamma image projected into the 3D LiDAR image of another contaminated environment described in [19].

Advances in stereo imaging and light or laser detecting and ranging (LiDAR) has enabled the development of systems [15,16] that can generate 3D visual maps of the environment. These systems have also been used as contextual sensors, in a passive sense, for radiation imaging. In [17,18], the authors demonstrated the effectiveness of combining a 3D design information verification (DIV) system with a Compton camera for nuclear safeguard applications. The 3D DIV system consisted mainly of a LiDAR system, dolly and associated algorithms that is able to generate high precision 3D visual maps of rooms and identify any modification or changes in object position [16]. The contaminated environment was simulated by two mock-up pipes (Figure 2a), one of which contained a line source (i.e., Europium-152). Integration of the images from both systems was accomplished by backprojecting the gamma image from the Compton camera into the 3D visual map. Consequently, objects and regions coincident with the gamma image are identified as contaminated (Figure 2b). In another similar experiment, the authors in [19] combined a coded aperture gamma camera and a LiDAR system for 3D radiation imaging. However, since the coded aperture camera provides no direction or depth information, a stereo camera was used as a bridge between the two systems. The stereo camera was attached to the gamma camera’s mask to align with the optical axis of the gamma camera so that the images from both cameras are automatically aligned. Then 3D points on the stereo image were then aligned to 3D points in the LiDAR image using software algorithms. After the alignment process, the 2D gamma image was then projected into the 3D image of the scene (Figure 2c).

Passive integration of radiological and contextual sensors described so far is relatively simple to implement and certainly gives a better understanding of the contaminated environment. However, simply overlaying or projecting the radiation image into the images generated by the contextual sensors does not fully account for the underlying dissimilarities between both systems especially in terms of resolution. This results in ambiguities as uncontaminated nearby objects or regions are indicated as contaminated, as seen in Figure 2b,c. Furthermore, such ambiguities will require human intervention to be resolved since the process is not quantified, thus preventing the automation of the process, which is very desirable in nuclear environments.

### 2.2. Active Integration of Radiological and Contextual Sensors

In order to fully harness the potential of contextual sensors for improved radiation detection/imaging, the data from these sensors need to play an active role in the final result of the combination process and not serve only as a passive backdrop over which the radiation image is overlaid. Such integration can be realised in the context of multi-sensor data fusion (MSDF). MSDF can be described as a well-defined organisation of sensors, data acquisition and processing techniques and decision support algorithms governed by a fusion architecture [20]. This enables the creation of a holistic view of an observation of interest and inferencing of additional information that will otherwise be impossible using only the individual sensors. The participating sensors in the fusion could be similar sensors measuring the same physical phenomenon from the same point in a kind of competitive fashion [21]. In such organisation, the goal of fusion is to increase the reliability and signal to noise ratio with the redundant data. On the contrary, the participating sensors can also be similar sensors measuring the same phenomenon, but from different points or even different types of sensors measuring different phenomena from different points [21]. Fusion of radiation and contextual sensors belong to this latter organisation where the complementary data from the multiple sensors are used to create a holistic understanding of the point of interest.

Furthermore, fusion of data from different sensors can be accomplished at broadly three different levels of data abstraction, namely: low (signal or pixel), medium (feature) and high (decision) levels [22,23]. Low level fusion techniques operate on the raw signal or in most cases on the image representations of the raw signals; hence, it is also referred to as signal- or pixel-level fusion. The output of pixel-level fusion is another image, which can be the input to higher fusion levels [23]. Medium- or feature-level fusion techniques operate on characteristic features extracted from the raw data. These features are representative of the observed physical phenomena and can be geometrical, statistical, structural or spectral features [23]. The extracted features are combined into a single feature vector to enable classification usually by automated reasoning algorithms that find correlations among the features [20]. In high- or decision-level fusion, the data from each sensor is fully processed to arrive at an intermediate decision about the phenomenon measured by that sensor. These decisions are then combined using automated reasoning algorithms to arrive at a global decision with a higher confidence [20]. It is important to note that feature and decision fusion levels are closely related, and it is a matter of design choice whether to fuse the extracted features before deriving a decision or to fuse the different decisions derived from the different features [23].

Finally, the MSDF of radiological and contextual sensors has been extensively researched and applied in the field of medical imaging where images from positron emission tomography (PET) and single photon emission computer tomography (SPECT) are fused with X-rays, ultrasound, magnetic resonance imaging (MRI), etc., images for improved diagnosis and localisation of diseased tissues [9]. These applications employ a plethora of techniques and algorithms that cuts across the three levels of MSDF. However, of interest in this paper is the relatively recent fusion of data from radiological and contextual sensors in non-medical applications [8,24,25,26,27,28,29]. Furthermore, since the interest is on the key role played by the contextual sensors in radiation detection and imaging, applications with the fusion of multiple datasets from only radiation detectors [30,31,32] are not included in the following review.

#### 2.2.1. Low Level Fusion of Radiological and Contextual Sensors

Most fusion techniques operate at this level because of time efficiency and avoidance of loss of information associated with higher fusion levels as a result of extracting only part of the raw data [23]. Consequently, most reported fusions of radiological and contextual sensors are low level fusions. In addition, these applications are dominated by the fusion of data from radiological and visual sensors and can be broadly divided into three application areas, namely: (1) detection and tracking of moving sources; (2) motion compensation of images of moving sources; (3) 3D volume fusion of radiation and visual images of the environment.

**Figure 3:**
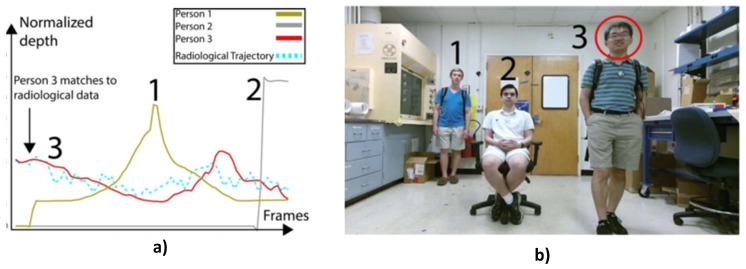
(**a**) Normalised depth trajectories of three targets and a radiation source (light blue); (**b**) image of targets with the suspect highlighted [24].

(1) Detection and tracking of moving sources:

The trajectories of radioactive sources in a security scenario are not predefined, e.g., a suspect carrying a radioactive source in a moving crowd. In addition, the trajectories also intersect with those of nearby objects and must be identified in real time with a minimum false alarm rate. A simple and low cost solution to this problem was proposed and demonstrated in [24]. The authors employed signal-/pixel-level fusion of a commercial depth sensor (Microsoft Kinect) and radiation detectors in order to track moving radiation sources. The method was based on extending traditional camera calibration [33] by attaching a radiation source to a chequerboard pattern and using the pattern to calibrate both sensors. This enabled the estimation of the radial distance of the radiation detector from the source using an integrated model of the detector’s count rate and the depth sensor’s calibration data. Consequently, the authors were able to track a source hidden amongst multiple moving targets by finding the target whose trajectory most closely matches the trajectory of the source (Figure 3).

**Figure 4:**
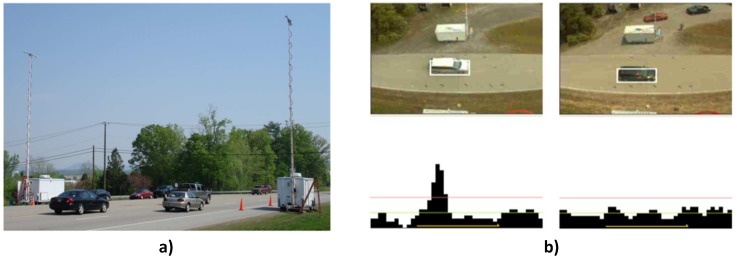
(**a**) Twin trailers. The video cameras were mounted on the top of the poles. (**b**) Top pictures are the images of the vehicle (bounded by white square), and the bottom graphs are the gamma count per pixel for a slice through the gamma image of the vehicle. The left images are for a vehicle with a source, and the right are for a vehicle without a source [26].

In another, but more elaborate solution, [26] combined a detector array of gamma imagers [34] with stereo cameras to demonstrate a portal-less highway radiation monitoring system. The classical radiation portal monitors are associated with several limitations amongst which are included: interference with the flow of traffic as vehicles need to slow down at the portals; ease of evasion by smugglers because the portal location is always fixed; and susceptibility to background variation caused by shielding of the detectors as vehicles pass in front of them [26]. Therefore, the portal-less system was designed to overcome these limitations. The system consisted of two identical sets of equipment fitted on two mobile trailers positioned on either side of the highway (Figure 4a). Vehicle detection and tracking algorithms [35] were applied to image frames from the video cameras to track the location of the vehicle from frame to frame as it traverses the camera’s field of view. These algorithms look for vehicle cues (e.g., regions with high gradients) that are consistent across these frames. After reconciliation of the video data from both trailers, the information was used to generate a sequence of video events that were in turn used to select the subset of gamma events from the gamma cameras that corresponds to the tracked vehicle. These gamma events were then used to generate a high statistic gamma image of the vehicle where radioactive sources are indicated by pixels whose significance is greater than a predefined threshold (Figure 4b). Results from field trials proved that the system was able to detect unshielded 27-MBq gamma point sources on five-lane highway traffic moving at speeds of up to 113 km/h. Finally, adaptation of this same system for autonomous radiation monitoring of small vessels in maritime environment was also reported in [25].

**Figure 5:**
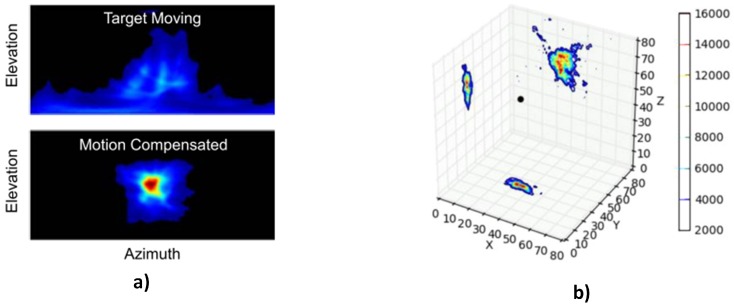
(**a**) Reconstructed image of moving source without (top) and with (bottom) motion compensation [27]; (**b**) 3D tomographic radiation source image on a stationary volume collapsed to the *x*-*y*, *y*-*z*, *x*-*z* planes [36].

(2) Motion compensation of images of moving sources:

Another challenge associated with imaging moving radioactive sources is the smearing of the source image across several pixels. This results in a blurred image where the source becomes indistinguishable from background radiation (Figure 5a). For instance, Compton imaging relies on the fact that Compton cones formed by photons from the same source will overlap coherently, thus forming a point in the image of higher intensity than the background. However, if the source is in motion, the apex of the cones end up in several pixels along the direction of motion; therefore, very few will overlap, resulting in the blurred image. A solution to this problem was demonstrated in [27] by combining video and Compton cameras. In the system, frames from the video cameras were analysed by a two-stage detection and tracking process in order to estimate the trajectory of the moving vehicle. The estimated trajectory was then used to adjust the position of the Compton cone during reconstruction so that the moving source appears stationary with respect to the detector, thus allowing the cones to overlap in a distinguishable hotspot on the image (Figure 5a).

The use of video camera image data to correct blurring of moving source images was also applied in [36] for ship to ship inspection in order to detect maritime smuggling of nuclear materials. The blurring of the image in this case is a result of wave action, which keeps both the inspection and target vessels in continuous motion. The key components of the system were a stereo video camera and a coded aperture gamma camera. Using both video and disparity images (which give the distance of points in the image from the camera plane) from the stereo camera, the relative location and orientation of both vessels were tracked in 3D across video frames. Using this information, a stationary reconstruction volume was then defined on a new coordinate system fixed at the target vessel. Voxels from this volume were then projected onto 2D images generated from the gamma camera from different position around the target vessel. Finally, gamma events from pixels in the 2D image closest to the centre of each of the projected voxels were accumulated into gamma events for that voxel resulting in a 3D tomographic radiation image (Figure 5b).

**Figure 6:**
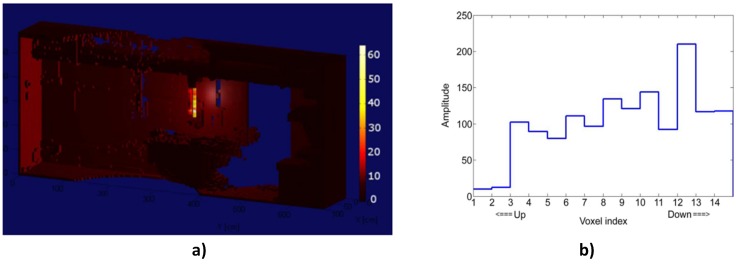
(**a**) Reconstructed image of contaminated environment shown in Figure 2a using the MSDF of the Compton camera and LiDAR images; (**b**) intensity of voxels from top to bottom around the pipe containing the line source [28].

(3) 3D volume fusion of the radiation image with the visual image of the environment:

Instead of simply projecting radiation images into already reconstructed 3D visual images of the environment, the data from both systems can be fully integrated during reconstruction to yield a 3D radiation image that is fused with the environment. This was demonstrated in [28] using a Compton camera and a 3D Design Information Verification system consisting mainly of a LiDAR system. First, the 3D data from the LiDAR were used to build a sparse 3D image space model divided into voxels. Then, backprojection weights were calculated for each photon event from the Compton camera image. Finally, a list-mode maximum likelihood algorithm [37] was then used to reconstruct the image by assigning weighted radiation intensity values to each voxel that was intersected by the surface of the Compton cone. This resulted in a spectroscopic 3D gamma-ray image of the scene, as shown in Figure 6a, which is the image of the same contaminated environment shown in Figure 2a. It can be clearly observed that the radioactive source is well localised in the pipe compared to the image in Figure 2b. The poor rendering of the surroundings is due to the use of a sparse image space in order to reduce computational resources. Figure 6b is a plot of the intensity of voxels around the pipe from top to bottom, which confirms that the gamma image is fused with the scene. Further application of this technique using other types of radiation cameras and Microsoft Kinect were also presented in [38].

#### 2.2.2. Higher Level Fusion of Radiological and Contextual Sensors

In general, the decision on the presence or otherwise of radioactive sources of interest in any radiation detection scheme is determined by the value of a threshold. The specific value of this threshold is derived from the measured data, and it defines the boundary above which the influence of naturally-occurring radiation (referred to as background radiation) is minimal. This is to ensure acceptable false alarm rates. However, large variations in background radiation across different environments increases uncertainty in the measurements (e.g., statistical noise), thereby making it difficult to select a suitable threshold value for a constant false alarm rate across different environments [39]. These variations are due to environmental factors, such as weather conditions, natural and man-made structures, e.g., roads, buildings, soil, etc. Furthermore, this variation cannot be well accounted for by assuming a Poisson background distribution [40]. A more robust solution will involve measuring background radiation in a variety of environments and finding correlations between the probability distribution of each environment with other environmental information, such as location, weather condition, material properties of both natural and man-made surrounding objects, etc. [8]. This implies the fusion of radiation data with data from a wide variety of sensors that measure these contextual factors in order to classify the background distributions from these environments. Furthermore, establishing meaningful relationship between datasets with such a high degree of heterogeneity is only possible at higher levels of data abstraction.

**Figure 7:**
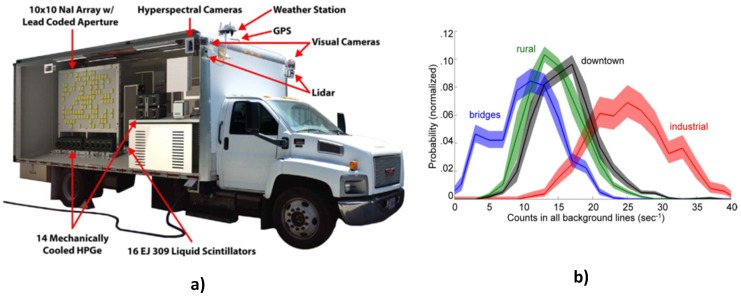
(**a**) Radiological Multi-sensor Analysis Platform (RadMap) [40]; (**b**) background radiation distribution classified by location [8].

Such an elaborate and exotic fusion described in the previous paragraph has been the objective of a series of research works [8,38,40,41] using the Radiological Multi-sensor Analysis Platform (RadMap). RadMap (Figure 7a) is a truck-mounted platform fitted with a variety of radiological and contextual sensors for large-scale acquisition of radiological and associated contextual data across different environments [40]. Results from a routine survey of districts in California [8] showed interesting correlations between identifiable features in the contextual data (i.e., places, objects, level of rainfall, etc.) and the background gamma spectra. For instance, Figure 7b shows the probability distribution of background radiation of four different environments categorised based on GPS locations. In addition, atmospheric pressure was confirmed to be the most dominant factor affecting variability in neutron background radiation [40]. This fusion of different features of the various datasets accurately captures the variability in the background radiation across different environments. Therefore, a detector system can simply adjust its detection threshold to the corresponding environment, thus maintaining a constant false alarm rate. Furthermore, such fused dataset can be used for advanced simulation of the performance of detector systems in real world using the concept of source injection where simulated radioactive sources are injected into real-world models of background radiation [41].

Another example of the high level fusion of radiological and contextual sensor was presented in [29] where decision-level fusion was used to integrate radiation and electromagnetic induction (EMI) data in order to detect and distinguish between buried depleted uranium (DU) and its oxide. Distinguishing between DU and DU oxide is important because DU oxides present a higher nuclear hazard; hence, their removal needs to be prioritised [29]. However, radiation detection techniques [42] are not able to distinguish between these two metals, hence the need for complementary information from the EMI data. First, the radiation data, collected over a gridded survey area, were processed by two anomaly detection algorithms. Each algorithm is able to come to an independent decision (i.e., radiation or non-radiation) about the presence or otherwise of a radiation target by using various background suppression methods. The quadrature components of the H-field measured by the EMI were analysed and compared with a database of known metals to produce three possible decisions, namely: DU metal, non-DU metal and non-metal.

**Figure 8:**
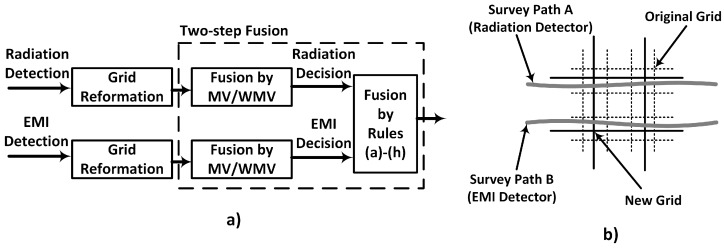
(**a**) Decision-level fusion framework for radiation and electromagnetic induction (EMI) data fusion (MV = mean vote, WMV = weighted mean vote); (**b**) re-gridding of the survey area [29].

Fusion of the decisions from both sensors followed a two-stage process (Figure 8a) after re-gridding the survey area so that mismatched survey paths of both systems can fall into the same cell (Figure 8b). The first stage was to fuse multiple radiation decisions from a single cell into one radiation decision and multiple EMI decisions from the same cell into one EMI decision. This was done using the maximum vote (MV) and weighted maximum vote (WMV) fusion methods [43]. The next stage combined the single decisions from each of the systems using eight fusion rules. These rules were if-then conditions that define the outcome for the desired combinations of both sets of decisions. For instance, two of the rules are: (1) if the EMI decision is “DU” and the radiation decision is “radiation”, then final decision is “DU”; (2) if the EMI decision is “non-DU metal” and the radiation decision is “radiation”, then the final decision is “oxide”. Experimental results confirmed the effectiveness of this fusion framework with over a 90% detection rate.

MSDF of radiological and contextual sensors at the pixel level yields high quality images with richer information content. However, the most significant advantage of MSDF is the quantification of the fusion process both at lower and higher levels. This enables the definition of metrics, such as root mean square error, entropy, fusion factor, etc. [44], for signal/pixel fusion and the receiver operator characteristic (ROC) for higher level fusion. These metrics provide an analytical basis for evaluating and optimising the outputs so as to reduce false alarm rates. Furthermore, arriving at a decision on the presence or otherwise of a radiation target in MSDF is inherently an automated process, thereby eliminating human errors. However, accurately calibrating and synchronizing the operations of the participating sensors in the fusion remains a challenging task. In addition, supervised classifiers used in higher level fusion require training with large datasets in order to perform at acceptable levels.

## 3. GPR as a Contextual Sensor for Radiological Characterisation of Nuclear Sites

Radiological characterisation involves the identification of the location, type and other physical properties of radioactive wastes. It is a critical stage in decommissioning potential nuclear contaminated sites and facilities as it provides input to other stages of the decommissioning process [45]. However, some of these wastes are often located in opaque and hard to access areas such as below the ground and inside concrete structures, as noted in Section 1. Traditional methods of characterising such wastes, e.g., gamma logging and core sampling, involve excavation, which leads to the generation of secondary wastes and increases the risks of exposure of personnel and equipment to ionising radiation. However, since these wastes are usually liquids or solids (i.e., the radioisotopes are usually attached to liquid or solid matter), contextual information about these liquids and solids (e.g., depth, volume, material type, etc.) inside these opaque areas can be obtained using non-intrusive geophysical methods. This contextual information can then be integrated with data from radiological sensors for enhanced non-intrusive characterisation of these wastes. The fusion of radiation and EMI sensors discussed in Section 2.2.2 tries to solve the case of radiation waste buried underground. However, EMI sensors can only detect metals and provide limited and unreliable target depth information [46]. Nevertheless, the successful fusion of both sensors shows the possibility of integrating radiological sensors with more advanced geophysical sensors like GPR.

**Figure 9:**
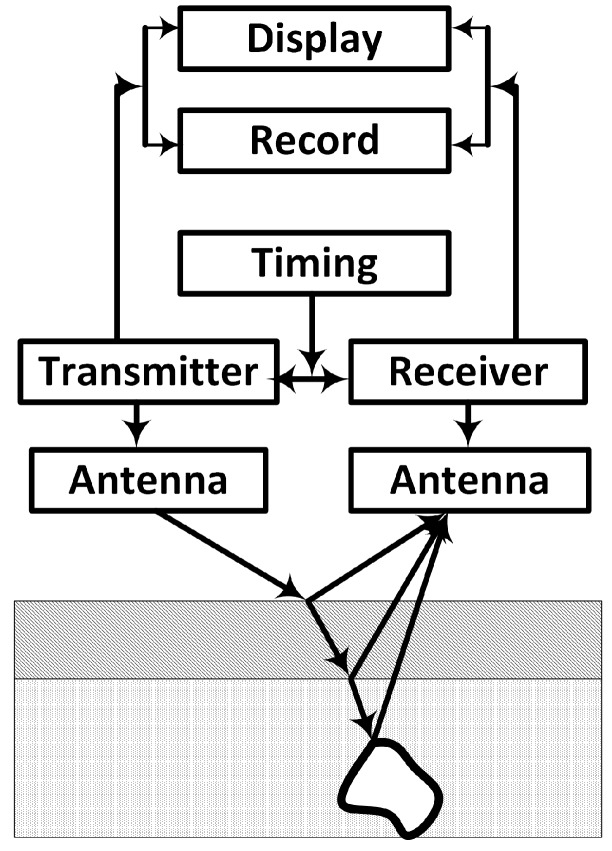
Block diagram of GPR operation showing reflections from different layers and an object underground. Adapted from [47].

GPR is a non-destructive geophysical technique for obtaining subsurface snapshots of visually opaque structures, thereby revealing hidden objects or internal property changes in the structure, such as defects and cavities. It is important to note that the term GPR is commonly used to refer to both the technique and the device. Figure 9 shows the block diagram of a typical GPR unit in a reflection survey. The transmitter generates a series of excitation voltages, which are transformed into a radiating electromagnetic (EM) field by the transmitting antenna. As the signal propagates through the subsurface, it undergoes different types of distortions, such as reflection, attenuation, etc., due to changes in the permittivity and conductivity of the subsurface. These changes are indicative of the presence of objects or discontinuities due to the transition from one material layer to another. The reflected signals are captured by the receiving antenna and processed in order extract useful information about the subsurface.

GPR is widely used for non-destructive subsurface investigation across a wide range of application areas [48]. However, of interest are: (1) non-destructive investigation of underground pipes; (2) non-destructive investigation of concrete structures. This is because these application areas are directly relevant to the challenges of characterising wastes in the identified hard to access areas. These application areas will further be discussed in the following subsections with the aim of highlighting where and how GPR and radiation detection/imaging can be integrated for radiological characterisation.

### 3.1. Non-Destructive Investigation of Underground Pipes

**Figure 10:**
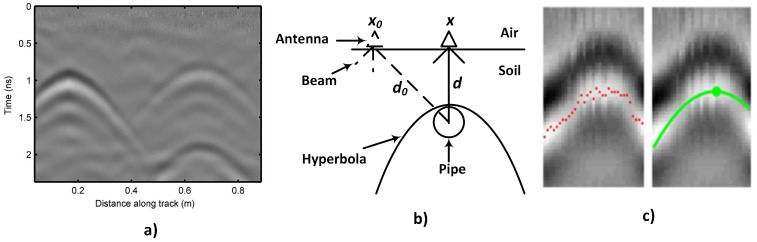
(**a**) GPR image showing three hyperbolas [59]; (**b**) formation of diffraction hyperbola; (**c**) hyperbola curve fitting [60].

Underground utility pipes and cables form a complex network of pipes, cables and drains in the subsurface [49]. This complexity in addition to their underground location makes their maintenance difficult without prior knowledge of their location. Furthermore, it has been shown that third party damages to utility pipes and cables during excavation is one of the major causes of increasing construction costs [50]. Consequently, GPR is widely used in location, classification and conditional assessment of underground pipelines [51,52,53]. Methods for locating buried pipes exploit the characteristic hyperbolas (Figure 10a) formed by buried objects in GPR radargrams. These hyperbolas are formed by the spreading of the EM waves (beam) as they leave the antenna, thereby illuminating the target before the target is directly under the antenna (Figure 10b). The equation for this hyperbola can be readily derived from Figure 10b (Equations (1) and (2)). It can be deduced from Equation (2) that by fitting synthetic hyperbolas to suitable points on a radargram (Figure 10c), the pipe’s radius, the wave velocity and the pipe’s depth can be estimated [54,55,56]. In addition, the retrieved wave velocity is an important parameter used in GPR reconstruction algorithms, such as matched filtering, backprojection, migration and tomographic inversion [57,58].

(1) $$d_{0}^{2}={\left(x-x_{0}\right)}^{2}+d^{2}$$

Substituting $d_{0}=v\times t_{0}/2+R$ and d=v×t/2+R into Equation (1) yields:

(2) $$\frac{{\left(t+\frac{2R}{v}\right)}^{2}}{{\left(t_{0}+\frac{2R}{v}\right)}^{2}}-\frac{{\left[2\left(x-x_{0}\right)\right]}^{2}}{{\left(\frac{vt_{0}}{2}+R\right)}^{2}}$$

Extensive pipeline networks, both underground and encased in concrete, are common features of nuclear facilities and are used for transporting liquid wastes [10]. Therefore, they constitute a significant amount of potential radioactive wastes that need to be characterised for effective decommissioning. However, their location in addition to inaccurate records makes their characterisation difficult. For instance, the UK has approximately 760 km of pipelines on nuclear sites out of which only about 26% have been characterised [10]. Typical detection of significant contamination in shallow buried pipes consists of a ground-level survey along the length of the pipe with a sensitive radiation detector [61]. However, the depth of the radiation source is required in order to know the intensity of the radiation at the pipe location, so as to implement the required safety measures before and during decommissioning. Furthermore, knowledge of the condition of the pipe is important so as not to destroy the pipe during excavation, thereby contaminating the surrounding soil. However, this contextual information is not available from a simple ground-level radiation measurement, thereby making pipeline decommissioning particularly challenging.

However, this required contextual information can be readily supplied by GPR. For instance, the depth of the pipes can be obtained from the GPR and used for 3D localisation of the contaminated hot spot (see Section 4). This can be used to guide automated systems, such as robots, to carry out the decommissioning operation. Furthermore, since the intensity of a radioactive source varies with the inverse of the square of the distance, a high level fusion framework can be design to integrate the radiation and GPR datasets to automatically identify and classify hot spots using the estimated radiation intensity. Such a system can be trained with historical data to be able to identify and distinguish significant contamination in pipes from background radiation in a variety of underground environments.

### 3.2. Non-Destructive Investigation of Concrete Structures

**Figure 11:**
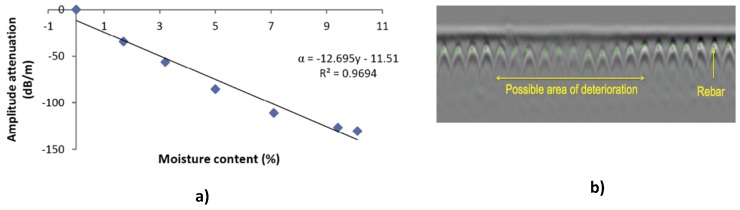
(**a**) GPR signal attenuation with varying moisture content in a concrete sample where α = attenuation (dB m^−1^), y = moisture content (%) and R^2^ = root mean square error [62]; (**b**) GPR image of concrete showing weak reflections from possibly corroded rebar [60].

GPR is widely used as a non-destructive technique in monitoring the health of concrete structures such as bridges, dams, tunnels, etc. Some of these applications include: moisture and chloride ingress; detection of rebar corrosion; and detection of voids and cracks [60,62,63]. GPR techniques for detecting moisture and chloride in concrete are based on energy loss by the GPR signals to water molecules and chloride ions due to increased conductivity. This is can be detected as an increase in the attenuation of the reflected signals (Figure 11a). GPR detection of concrete rebar corrosion makes extensive use of hyperbola detection techniques described in Section 3.1. This is because reflections from corroded rebar are weaker than reflections from non-corroded rebar and are displayed as lighter hyperbola traces in GPR images (Figure 11b). This information can then be processed to produce a deterioration map of the concrete structure [60].

Concrete is a ubiquitous construction material used extensively in nuclear sites due to its shielding ability [11,64]. Therefore, it is the main component of many structures, such as reactor containments and biological shields at nuclear sites. Contamination of these concrete structures is as a result irradiation from fission products and from leaks and spills of liquid contaminants during operations [45]. Furthermore, interaction between the concrete and these contaminants results in defects [64], thereby enabling the contamination to penetrate deep into the concrete, and can even lead to neutron activation of the concrete rebar. Decontamination of concrete for free release is done by removing the contaminated layers using methods such as scarification [11]. However, without knowledge of the depth of the contamination, the amount of material to be removed can only be known by continuous radiation survey after each pass of the equipment, which is expensive and time wasting. In addition, if the contamination is beyond certain depths, it is usually more cost effective to adopt complete removal of the structure rather than scarifying [45].

The use of GPR to detect the state of the rebar might be a useful technique for estimating the depth of contamination. However, this assumes that the contaminant has penetrated to the rebar, which is not always true. A more robust solution is to analyse the changes in the electrical properties (i.e., permittivity and conductivity) of concrete as a result of the presence of radioactive sources and finding correlations between these changes and reflected GPR signals. This is because GPR signals are responsive to changes in the electrical properties of the medium in which they propagate. These correlations can then serve as a basis for fusing the GPR and radiation data in order to develop new techniques for non-destructive characterisation of internal contamination in concrete structures.

## 4. Combined GPR and Radiation Imaging for 3D Localisation of Radioactive Contamination in Underground Pipes

Localisation of radiation sources in 3D space is an important part of radiological characterisation. This section uses MCNPX (Version 2.7) [65] and gprMax (Version 3.0) [66] modelling and simulations to demonstrate the inadequacy of ground-level radiation imaging for localising underground contamination and how additional information from GPR can help to localise the contamination in 3D, thereby resolving ambiguities.

### 4.1. Methodology

**Figure 12:**
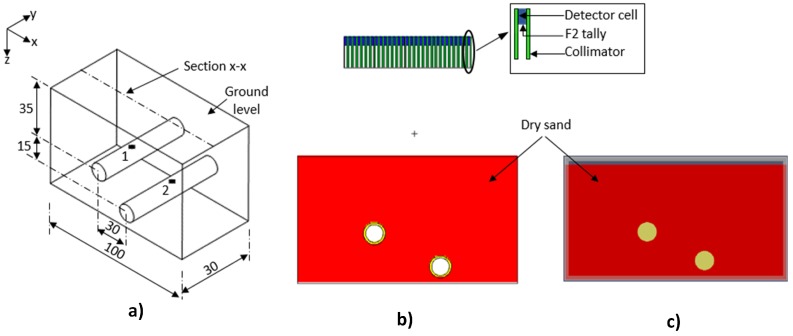
(**a**) Modelled contaminated environment (dimensions in cm); (**b**) MCNPX model with grid of detectors (section x-x); (**c**) gprMax model (section x-x).

The modelled environment (Figure 12a) is a section of two underground pipes used for transporting liquid radioactive wastes. Both pipes are separated vertically and horizontally with contamination located at their crown beneath the points (1 and 2) indicated in the figure. This type of internal contamination is mostly due to scale build up on the internal surfaces of pipes or accumulation of sludge and silt in pipes and can be detected at the ground surface for shallow buried pipes [10,61]. The MCNPX model (Figure 12b) consisted of two cast iron pipes (internal radius = 4 cm, external radius = 5 cm, density = 7.15 g cm^−3^) buried in dry sand (density = 1.7 g cm^−3^). The contaminated points were modelled as Co-60 point sources (a common radioactive contaminant in pipes [10]) with relative strengths of 0.15 and 0.85 for Sources 1 and 2, respectively. In gprMax, the environment was modelled as a 2D slice along section x-x, which is centred at the *y*-axis (Figure 12c). A 2D GPR model was used because the modelled environment is symmetrical about the *x*-axis. Furthermore, the pipes were modelled as two perfect electrical conducting cylinders buried in dry sand (relative permittivity = 3.89).

The radiation image was acquired by a 29 × 15 grid of detectors (Figure 12b) placed 40 cm above the ground and centred at the *x-y* plane. This is equivalent to moving a single detector in 29 × 15 discrete positions on the ground surface. This number of grids was chosen because they were enough to cover the area occupied by both pipes. Each detector (Figure 12b inset) consisted of a cylindrical cell (radius = 0.5 cm and height = 3 cm) surrounded by 0.5 cm-thick tungsten collimator, which is 14 cm long. The MNCPX F2 tally was placed at the bottom of the detector cell to record all gamma photon events at that surface. The GPR data were obtained by a collocated transmitter-receiver Hertzian dipole pair at 256 locations along section x-x. It should be noted that in practice, the path along which to acquire the GPR measurements can be determined from the acquired radiation image. Furthermore, the GPR transmitter was excited with a Gaussian wavelet centred at 1 GHz because of the relatively shallow depth of the pipes. In order to minimise dispersion errors, the GPR simulation used a spatial resolution of Δx = Δy = 0.002 m, which is less than one tenth of the smallest wavelength present in the model. Finally, the temporal resolution Δt was calculated to be 4.7 ps using the Courant, Freidrichs and Lewy stability condition given by Equation (3), where *c* is the wave velocity in free space.

(3) $$\Delta t\leq \frac{1}{c\sqrt[{}]{\frac{1}{\Delta x^{2}}+\frac{1}{\Delta y^{2}}}}$$

#### 4.1.1. Matched Filter Synthetic Aperture Radar Imaging

**Figure 13:**
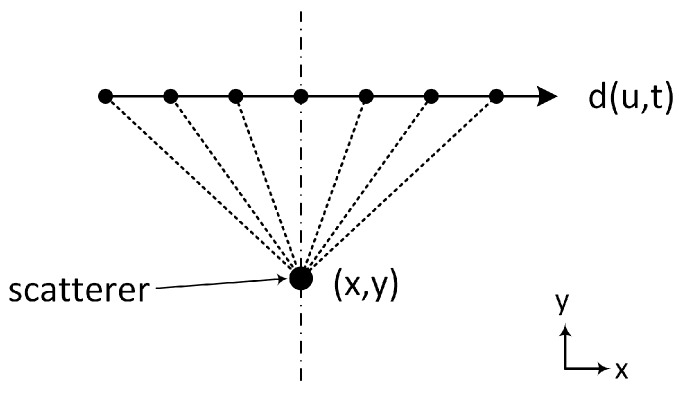
Monostatic linear SAR data collection.

The GPR data were processed using the matched filter SAR imaging algorithm [57]. Consider the monostatic linear collection scenario in Figure 13. The data d(u,t) collected along the cross range are the time delayed, amplitude scaled version of the point spread response (PSR) $s(u,t-t_{d})$ of the stationary scatterer at (x,y) where the time delay $t_{d}$ is given by Equation (4) (*v* is the wave velocity in the medium). However, for a signal corrupted with additive white Gaussian noise, the optimum match filter is the conjugate of the time reversed version of the signal of interest [57]. Therefore, the matched filtered output at a given location is given by the convolution of the collected data and the conjugate of the PSR at that location. This convolution operation is given by Equation (5).

(4) $$t_{d}=\frac{2}{v}\sqrt[{}]{{\left(u-x\right)}^{2}+y^{2}}$$

(5) $$I\left(x,y\right)=d\left(u,t\right)*s^{*}\left(u,t-t_{d}\right)=\int_{-\infty }^{\infty }\int_{-\infty }^{\infty }d\left(u,t\right)s^{*}\left(u,t-t_{d}\right)dudt$$

In matched filter SAR imaging, the PSR is calculated for each pixel and applied to the collected data using Equation (5). This results in an image where pixels in the location of scatterers have higher signal to noise ratios compared to surrounding pixels. Finally, even though the matched filter SAR algorithm requires significant processing power for large datasets, it is however robust and has practical applications for small and moderate datasets.

### 4.2. Simulation Results

**Figure 14:**
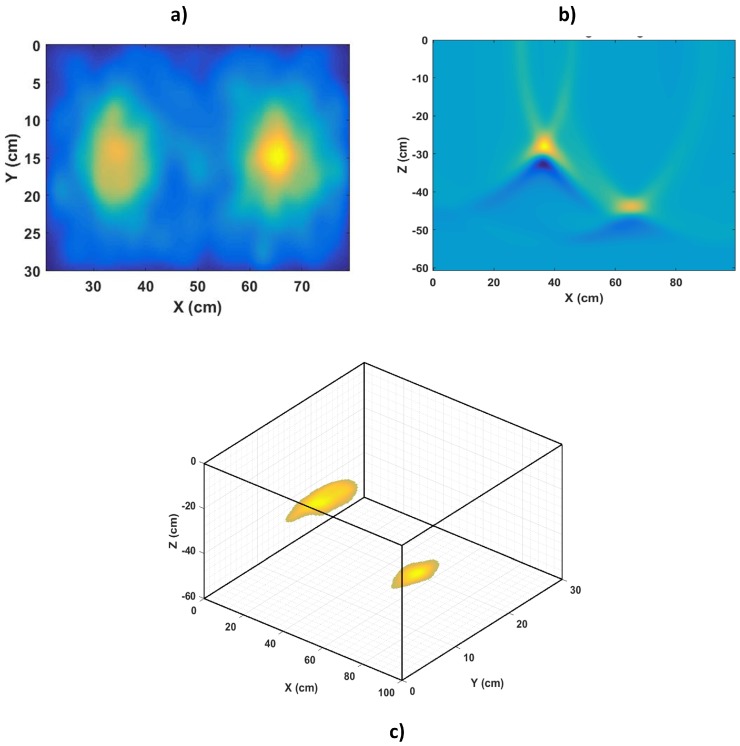
(**a**) Ground level image of radiation sources; (**b**) matched filtered GPR image of pipes; (**c**) 3D localised image of radiation sources.

The radiation image is shown in Figure 14a after resampling and smoothing with a 25 × 25 pixel Gaussian window [3]. As expected, the vertical separation between the two pipes is not observable in the radiation image because of the lack of depth information. Furthermore, the fact that Source 2 is measured as having a higher intensity could easily be misconstrued to imply that Source 2 is closer to the surface than Source 1. Figure 14b shows the GPR image after processing with the matched filter SAR imaging algorithm described in Section 4.1.1. This yields the required depth information, as it shows the points where the GPR signals were reflected by the pipe surfaces. Using this depth information, the radiation image can be projected into the ground to yield a 3D localised radiation image (Figure 14c), thereby resolving any ambiguity associated with the lack of depth information. Note that only pixels with normalised intensity ≥0.7 for both sources were projected back to the pipe locations. This integration of GPR and radiation imaging is similar to the passive integration methods described in Section 2.1 where the radiation image is projected into a 3D visual image of the environment. However, the use of GPR rather than visual sensors enabled the detection of the pipe beyond the ground surface, thereby allowing the contaminated spot to be localised on the pipe surfaces. Furthermore, this 3D image can be used to guide automated systems, such as robots, during decommissioning of the contaminated pipes.

However, it must be noted that these simulations assume an idealised scenario where the effects of noise, both from background radiation and clutter in the radiation and radar images, respectively, were not considered. However, techniques for dealing with noise encountered both in radiation and radar images from the field have been presented in the literature [29,56]. In addition, the retrieved depth of the contamination assumes that each pipe’s thickness is negligible so that the distances from the ground surface to both the pipe’s surface and the pipe’s crown (actual location of the contamination) are approximately the same. This is a practical assumption as the thickness of typical metallic and PVC pipes used in nuclear pipelines are between 5 and 12 mm [10]. Finally, another potential improvement is the simultaneous reconstruction of the GPR and radiation data at the point of reflection (i.e., the pipe surfaces). This will require acquisition of GPR measurements at multiple slices along the *y*-axis (i.e., 3D GPR data) so that points in the radiation data that correlate with points in the GPR data can be backprojected during reconstruction of the GPR image. The output of such an algorithm will be a 3D fused image of the radiation and GPR data at the pipe surface similar to the output of the 3D volume fusion technique described in Section 2.2.1.

## 5. Conclusions

Recent applications of integrated radiological and contextual sensors techniques for detecting and localising radioactive sources in non-medical field have been reviewed. The use of contextual sensors enabled the retrieval of additional contextual information about the radiation source, thereby enabling characterisation of radioactive sources in challenging scenarios. However, these applications are dominated by the use of visual sensors as contextual sensors. Furthermore, visual sensors cannot retrieve contextual information about radioactive wastes in visually-inaccessible locations typically encountered at nuclear sites and facilities. Therefore, GPR was proposed and examined as a contextual sensor for characterising such wastes in light of the techniques presented in the reviewed literature.

Furthermore, it was identified that integration of GPR and radiological sensors will potentially enable non-intrusive characterisation of radioactive wastes in nuclear sites and facilities especially in two critical areas namely: (1) contaminated pipelines buried underground or encased in concrete and (2) contamination ingress into porous materials, such as concrete. This will enable rapid characterisation of wastes in these hard to access areas while reducing dosage risks and the generation of secondary wastes. However, more research is required in order to understand and identify correlating attributes in both GPR and radiation datasets acquired from these contaminated environments. This is because multi-sensor integration techniques rely on establishing relationships among the data from the participating sensors.

Finally, the presented simulation results have demonstrated the effectiveness of combined GPR and radiation imaging for 3D localisation of contamination in buried pipelines by projecting the radiation image back to the pipe location. 3D localisation is an important part of the characterisation process, and such 3D localised images can be used to guide automated systems, such as robots, during decommissioning of the contaminated pipes. However, a 3D reconstruction algorithm needs to be developed in order to enable simultaneous reconstruction of the radiation and GPR data at the point of reflection. This will yield a fused radiation and GPR image with improved localisation of the contaminated region compared to simply projecting the radiation image back to the location of the pipes. Furthermore, the implementation of the reconstruction algorithm can take advantage of available fast microprocessors to enable real-time reconstruction of the underground contaminated environment.

## Abbreviations

The following abbreviations are used in this manuscript:

GPR	Ground-penetrating radar
MSDF	Multi-sensor data fusion

## References

[1] Elkins K.C., Moncayo V.M., Kim H., Olson L.D. (2017). Utility of gray-matter segmentation of ictal-Interictal perfusion SPECT and interictal 18F-FDG-PET in medically refractory epilepsy. Epilepsy Res..

[2] Clifford E.T.H., McFee J.E., Ing H., Andrews H.R., Tennant D., Harper E., Faust A.A. (2007). A militarily fielded thermal neutron activation sensor for landmine detection. Nucl. Instrum. Methods Phys. Res. Sect. A Accel. Spectrom. Detect. Assoc. Equip..

[3] Gal O., Dessus B., Jean F., Lainé F., Lévêque C. (2001). Operation of the CARTOGAM portable gamma camera in a photon-counting mode. IEEE Trans. Nucl. Sci..

[4] Carrel F., Khalil R.A., Colas S., Toro D.D., Ferrand G., Gaillard E., Gmar M., Hameau D., Jahan S., Lainé F. GAMPIX: A New Gamma Imaging System for Radiological Safety and Homeland Security Purposes. Proceedings of the 2011 IEEE Nuclear Science Symposium and Medical Imaging Conference (NSS/MIC).

[5] Wahl C.G., Kaye W.R., Wang W., Zhang F., Jaworski J.M., King A., Boucher Y.A., He Z. (2015). The Polaris-H imaging spectrometer. Nucl. Instrum. Methods Phys. Res. Sect. A Accel. Spectrom. Detect. Assoc. Equip..

[6] Gamage K., Joyce M., Adams J. (2011). Combined digital imaging of mixed-field radioactivity with a single detector. Nucl. Instrum. Methods Phys. Res. Sect. A Accel. Spectrom. Detect. Assoc. Equip..

[7] Knoll G. (2010). Radiation Interactions. Radiation Detection and Measurement.

[8] Aucott T.J., Bandstra M.S., Negut V., Chivers D.H., Cooper R.J., Vetter K. (2013). Routine surveys for Gamma-Ray background characterization. IEEE Trans. Nucl. Sci..

[9] James A.P., Dasarathy B.V. (2014). Medical image fusion: A survey of the state of the art. Inf. Fusion.

[10] Miller B., Foster A., Nuvia M.D., Hill M., Foster A. (2016). Pipeline Characterisation and Decommissioning within the Nuclear Industry: Technology Review and Site Experience.

[11] Sullivan P.O., Nokhamzon J.G., Cantrel E. (2010). Decontamination and dismantling of radioactive concrete structures. NEA News.

[12] Gmar M., Gal O., Goaller C.L., Ivanov O.P., Potapov V.N., Stepanov V.E., Laine F., Lamadie F. (2003). Development of coded-aperture imaging with a compact gamma camera. IEEE Trans. Nucl. Sci..

[13] Gal O., Gmar M., Ivanov O.P., Lainé F., Lamadie F., Le Goaller C., Mahé C., Manach E., Stepanov V.E. (2006). Development of a portable gamma camera with coded aperture. Nucl. Instrum. Methods Phys. Res. Sect. A Accel. Spectrom. Detect. Assoc. Equip..

[14] Hughes K., Lightfoot J. RadScan 600-a portable instrument for the remote imaging of gamma contamination: Its design and use in aiding decommissioning strategy. Proceedings of the 1996 IEEE Nuclear Science Symposium Conference Record.

[15] Ecker M., Vincent R. (2010). Light Detection and Ranging ( LiDAR ) Technology Evaluation.

[16] Sequeira V., Goncalves J.G.M. (2003). 3D Verification of Plant Design. Proceedings of the 25th ESARDA Symposium on Safeguards and Nuclear Materials Management.

[17] Mihailescu L., Vetter K., Ruhter W., Chivers D., Coates C., Smith S., Hines J., Caiado A.C.R., Sequeira V., Fiocco M. (2006). Combined Measurements with Three-Dimensional Design Information Verification System and Gamma Ray Imaging—A Collaborative Effort Between Oak Ridge National Laboratory, the Joint Research Center at Ispra. Proceedings of the 47th INMM Annual Meeting.

[18] Raffo-Caiado A.C., Ziock K.P., Hayward J.P., Smith S., Solodov A., Mihailescu L., Vetter K., Dougan A., Burks M., Goncallves J. (2009). Investigation of Combined Measurements with Three-Dimensional Design Information Verification System and Gamma-Ray Imaging Systems for International Safeguards Applications. Proceedings of the 50th INMM Annual Meeting.

[19] Boehnen C., Paquit V., Ziock K., Guzzardo T., Whitaker M., Raffo-Caiado A. Field trial of a highly portable coded aperture gamma ray and 3D imaging system. Proceedings of the 2011 Future of Instrumentation International Workshop (FIIW).

[20] Hall D., Llinas J. (2002). An introduction to multisensor data fusion. Proc. IEEE.

[21] Durrant-Whyte H.F. (1988). Sensor Models and Multisensor Integration. Int. J. Rob. Res..

[22] Luo R., Kay M. (1992). Data Fusion and Sensor Integration.

[23] Basaeed E., Bhaskar H., Al-Mualla M. Beyond pan-sharpening: Pixel-level fusion in remote sensing applications. Proceedings of the 2012 IEEE International Conference on Innovations in Information Technology (IIT).

[24] Riley P., Enqvist A., Koppal S.J. Low-Cost Depth and Radiological Sensor Fusion to Detect Moving Sources. Proceedings of the 2015 IEEE International Conference on 3D Vision (3DV).

[25] Ziock K.P., Cheriyadat A., Fabris L., Goddard J., Hornback D., Karnowski T., Kerekes R., Newby J. (2011). Autonomous radiation monitoring of small vessels. Nucl. Instrum. Methods Phys. Res. Sect. A Accel. Spectrom. Detect. Assoc. Equip..

[26] Ziock K.P., Bradley E.C., Cheriyadat A., Cunningham M., Fabris L., Fitzgerald C.L., Goddard J.S., Hornback D.E., Kerekes R.A., Karnowski T.P. (2013). Performance of the roadside tracker portal-less portal monitor. IEEE Trans. Nucl. Sci..

[27] Gao D., Yao Y., Pan F., Yu T., Yu B., Guan L., Dixon W., Yanoff B., Tian T.P., Krahnstoever N. Computer vision aided target linked radiation imaging. Proceedings of the 2012 IEEE Conference on Computer Vision and Pattern Recognition (CVPR).

[28] Mihailescu L., Vetter K., Chivers D. (2009). Standoff 3D gamma-ray imaging. IEEE Trans. Nucl. Sci..

[29] Long Z., Wei W., Turlapaty A., Du Q., Younan N.H. (2013). Fusion of radiation and electromagnetic induction data for buried radioactive target detection and characterization. IEEE Trans. Nucl. Sci..

[30] Sundaresan A., Varshney P.K., Rao N.S.V. Distributed detection of a nuclear radioactive source using fusion of correlated decisions. Proceedings of the 2007 IEEE 10th International Conference on Information Fusion.

[31] Chin J.C., Yau D.K., Rao N.S., Yang Y., Ma C.Y., Shankar M. Accurate localization of low-level radioactive source under noise and measurement errors. Proceedings of the 6th ACM conference on Embedded Network Sensor Systems.

[32] Rao N.S.V., Sen S., Prins N.J., Cooper D.A., Ledoux R.J., Costales J.B., Kamieniecki K., Korbly S.E., Thompson J.K., Batcheler J. (2015). Network algorithms for detection of radiation sources. Nucl. Instrum. Methods Phys. Res. Sect. A Accel. Spectrom. Detect. Assoc. Equip..

[33] Moreno D., Taubin G. Simple, accurate, and robust projector-camera calibration. Proceedings of the 2012 IEEE Second International Conference on 3D Imaging, Modeling, Processing, Visualization and Transmission (3DIMPVT).

[34] Ziock K.P., Fabris L., Carr D., Collins J., Cunningham M., Habte F., Karnowski T., Marchant W. (2008). A fieldable-prototype, large-area, gamma-ray imager for orphan source search. IEEE Trans. Nucl. Sci..

[35] Lucas B.D., Kanade T. An iterative image registration technique with an application to stereo vision. Proceedings of the 7th international joint conference on Artificial intelligence.

[36] Ziock K.P., Boehnen C.B., Ernst J.M., Fabris L., Hayward J.P., Karnowski T.P., Paquit V.C., Patlolla D.R., Trombino D.G. (2016). Motion correction for passive radiation imaging of small vessels in ship-to-ship inspections. Nucl. Instrum. Methods Phys. Res. Sect. A Accel. Spectrom. Detect. Assoc. Equip..

[37] Wilderman S.J., Fessler J.A., Clinthorne N.H., LeBlanc J.W., Rogers W.L. (2001). Improved modeling of system response in list mode EM reconstruction of Compton scatter camera images. IEEE Trans. Nucl. Sci..

[38] Vetter K. (2016). Multi-sensor radiation detection, imaging, and fusion. Nucl. Instrum. Methods Phys. Res. Sect. A Accel. Spectrom. Detect. Assoc. Equip..

[39] Ziock K.P., Collins J.W., Fabris L., Gallagher S., Horn B.K.P., Lanza R.C., Madden N.W. (2006). Source-Search Sensitivity of a Large-Area, Coded-Aperture, Gamma-ray Imager. IEEE Trans. Nucl. Sci..

[40] Bandstra M.S., Aucott T.J., Brubaker E., Chivers D.H., Cooper R.J., Curtis J.C., Davis J.R., Joshi T.H., Kua J., Meyer R. (2016). RadMAP: The Radiological Multi-sensor Analysis Platform. Nucl. Instrum. Methods Phys. Res. Sect. A Accel. Spectrom. Detect. Assoc. Equip..

[41] Aucott T.J., Bandstra M.S., Negut V., Curtis J.C., Chivers D.H., Vetter K. (2014). Effects of Background on Gamma-Ray Detection for Mobile Spectroscopy and Imaging Systems. IEEE Trans. Nucl. Sci..

[42] Du Q., Wei W., May D., Younan N.H. (2010). Noise-adjusted principal component analysis for buried radioactive target detection and classification. IEEE Trans. Nucl. Sci..

[43] Yang H., Du Q., Ma B. (2010). Decision fusion on supervised and unsupervised classifiers for hyperspectral imagery. IEEE Geosci. Remote Sens. Lett..

[44] Galande A., Patil R. The Art of Medical Image Fusion: A Survey. Proceedings of the 2013 IEEE International Conference on Advances in Computing, Communications and Informatics (ICACCI).

[45] Laraia M.T. (2012). Nuclear Decommissioning: Planning, Execution and International Experience.

[46] Abdel-Aleem M., Chibelushi C.C., Moniri M. Multisensor data fusion for the simultaneous location and condition assessment of underground water pipelines. Proceedings of the 2011 IEEE International Conference on Networking, Sensing and Control (ICNSC).

[47] Annan A.P., Jol H.M. (2009). Electromagnetic Principles of Ground Penetrating Radar. Ground Penetrating Radar Theory and Applications.

[48] Daniels D.J., Daniels D.J. (2004). Properties of Materials. Ground Penetrating Rada.

[49] Rogers C.D.F. Support Smart and Liveable Cities. Proceedings of the 22015 IEEE 8th International Workshop on Advanced Ground Penetrating Radar (IWAGPR).

[50] Metje N., Ahmad B., Crossland S.M. (2015). Causes, impacts and costs of strikes on buried utility assets. Proc. Inst. Civ. Eng. Munici. Eng..

[51] Mooney J.P., Ciampa J.D., Young G.N., Kressner A.R., Carbonara J. GPR mapping to avoid utility conflicts prior to construction of the M-29 transmission line. Proceedings of the 2010 IEEE PES Transmission and Distribution Conference and Exposition.

[52] El-Mahallawy M.S., Hashim M. (2013). Material classification of underground utilities from GPR images using DCT-based SVM approach. IEEE Geosci. Remote Sens. Lett..

[53] Ayala-Cabrera D., Herrera M., Izquierdo J., Ocaña-Levario S.J., Pérez-García R. (2013). GPR-based water leak models in water distribution systems. Sensors.

[54] Shihab S., Al-Nuaimy W. (2005). Radius estimation for cylindrical objects detected by ground penetrating radar. Subsurf. Sens. Technol. Appl..

[55] Qiao L., Qin Y., Ren X., Wang Q. (2015). Identification of Buried Objects in GPR Using Amplitude Modulated Signals Extracted from Multiresolution Monogenic Signal Analysis. Sensors.

[56] Mertens L., Persico R., Matera L., Lambot S. (2016). Automated Detection of Reflection Hyperbolas in Complex GPR Images with No. a Priori Knowledge on the Medium. IEEE Trans. Geosci. Remote Sens..

[57] Richards M.A., Scheer J.A., Holm W.A. (2010). Matched Filter Imaging. Principles of Modern Radar Vol. I: Basic Principles.

[58] Gonzalez-Huici M.A., Catapano I., Soldovieri F. (2014). A comparative study of GPR reconstruction approaches for landmine detection. IEEE J. Sel. Top. Appl. Earth Obs. Remote Sens..

[59] Huuskonen-Snicker E., Mikhnev V.A., Olkkonen M.K. (2015). Discrimination of buried objects in impulse GPR using phase retrieval technique. IEEE Trans. Geosci. Remote Sens..

[60] Kaur P., Dana K.J., Romero F.A., Gucunski N. (2015). Automated GPR Rebar Analysis for Robotic Bridge Deck Evaluation. IEEE Trans. Cybern..

[61] Miller B., Foster A., Burgess P., Metrology R., Hill M., Foster A. (2016). Pipeline Characterisation and Decommissioning within the Nuclear Industry: Good Practice Guide.

[62] Senin S., Hamid R. (2016). Ground penetrating radar wave attenuation models for estimation of moisture and chloride content in concrete slab. Constr. Build. Mater..

[63] Trela C., Kind T., Schubert M., Gunther M., Eichen U.D., Trelabamde C. Detection of Weak Scatterers in Reinforced Concrete Structures. Proceedings of the 2014 IEEE 15th International Conference on Ground Penetrating Radar (GPR).

[64] Norris W.E., Naus D.J., Graves H.L. (1999). Inspection of nuclear power plant containment structures. Nucl. Eng. Des..

[65] Pelowitz D.B. (2011). MCNPX User’s Manual, Version 2.7.0.

[66] Warren C., Giannopoulos A., Giannakis I. (2016). gprMax: Open source software to simulate electromagnetic wave propagation for Ground Penetrating Radar. Comput. Phys. Commun..

